# The association of SOD and HsCRP with the efficacy of sulforaphane in schizophrenia patients with residual negative symptoms

**DOI:** 10.1007/s00406-023-01679-7

**Published:** 2023-09-20

**Authors:** Jianfei Zeng, Weizhi Zhang, Xiaobing Lu, Hui Zhou, Jing Huang, Zhenyu Xu, Hairong Liao, Jiaquan Liang, Meihong Liang, Chan Ye, Ting Sun, Yutong Hu, Qi She, Haixia Chen, Qian Guo, LiuJiao Yan, Renrong Wu, Zezhi Li

**Affiliations:** 1 Department of Nutritional and Metabolic Psychiatry, The Affiliated Brain Hospital of Guangzhou Medical University, Mingxin Road #36, Liwan District, Guangzhou, 510370 China; 2 Shenzhen Mental Health Center/Shenzhen Kangning Hospital, Shenzhen, China; 3 Guangdong Engineering Technology Research Center for Translational Medicine of Mental Disorders, Guangzhou, China; 4 Shiyan People’s Hospital of Baoan District, Shenzhen, China; 5 Department of Psychiatry, National Clinical Research Center for Mental Diseases, The Second Xiangya Hospital of Central South University, Changsha, China; 6 Ganzhou People’s Hospital of Jiangxi Province, Ganzhou, China; 7 The Third People’s Hospital of Foshan, Foshan, China; 8 University of Chinese Academy of Science-Shenzhen Hospital, Shenzhen, China; 9 Zhuhai Center for Chronic Disease Control/The Third People’s Hospital of Zhuhai, Zhuhai, China; 10 Zhongshan Third People’s Hospital, Zhongshan, China; 11 Zhaoqing Third People’s Hospital, Zhaoqing, China; 12 Key Laboratory of Neurogenetics and Channelopathies of Guangdong Province and the Ministry of Education of China, Guangzhou Medical University, Guangzhou, China; 13 Jiangsu Key Laboratory of Neurodegeneration, Nanjing Medical University, Nanjing, China

**Keywords:** Schizophrenia, Sulforaphane, Negative symptoms, High sensitivity C-reactive protein, Superoxide dismutase

## Abstract

**Objectives:**

Emerging evidence indicates a connection between oxidative stress, immune-inflammatory processes, and the negative symptoms of schizophrenia. In addition to possessing potent antioxidant and anti-inflammatory properties, sulforaphane (SFN) has shown promise in enhancing cognitive function among individuals with schizophrenia. This study aims to investigate the efficacy of combined treatment with SFN in patients with schizophrenia who experience negative symptoms and its effect on the levels of superoxide dismutase (SOD) and the inflammatory marker, high-sensitivity C-reactive protein (HsCRP).

**Design:**

Forty-five patients with schizophrenia were recruited, who mainly experienced negative symptoms during a stable period. In addition to the original treatments, the patients received SFN tablets at a daily dose of 90 mg for 24 weeks. At baseline, 12 weeks, and 24 weeks, the participants were interviewed and evaluated. The reduction rate of the Positive and Negative Syndrome Scale (PANSS) was used to assess each participant. The side effects scale of Treatment Emergent Symptom Scale (TESS) was applied to assess the adverse reactions. Additionally, the levels of the SOD, HsCRP, and other indicators were examined.

**Results:**

The study findings revealed a significant decrease in PANSS negative subscale scores (*P* < 0.001). Furthermore, there was a significant increase in SOD activity and HsCRP levels (*P* < 0.001 and *P* < 0.05). Notably, the group of participants who exhibited a reduction in PANSS negative subscale scores demonstrated a significant improvement in HsCRP levels (*P* < 0.05).

**Conclusions:**

Our study suggests that SFN may potentially serve as a safe adjunctive intervention to improve the negative symptoms of schizophrenia. The potential mechanism by which SFN improves negative symptoms in schizophrenia patients may involve its anti-inflammatory properties, specifically its ability to reduce HsCRP levels.

Trial registration

ClinicalTrial.gov (ID: NCT03451734).

**Supplementary Information:**

The online version contains supplementary material available at 10.1007/s00406-023-01679-7.

## Introduction

Schizophrenia is a chronic and severe psychiatric disorder primarily characterized by the presence of positive and negative symptoms, cognitive impairment, and social dysfunction [1]. Although the antipsychotic agents are available, there are still a portion of patients who have poor response to the antipsychotics, and even develop into treatment-resistant schizophrenia [2]. In particular, patients with treatment-resistant negative symptoms experience a poor outcome, and their social function is significantly impaired [3, 4]. Although the mechanisms of schizophrenia is still unclear, an increasing body of evidence suggests that immunoinflammatory processes and oxidative stress play a key role in the in the mechanisms of negative symptoms observed in schizophrenia [5, 6]. Numerous studies have demonstrated a strong association between immune cell dysregulation and abnormalities in pro-inflammatory cytokines with both the severity and improvement of negative symptoms [7, 8]. The infiltration of cytokines, including tumor necrosis factor (TNF), interleukin 1 (IL-1), and interleukin 6 (IL-6), into the brain alongside the acute phase reactant C-reactive protein (CRP) can result in tissue damage, neurodegeneration, reduced activation of the ventral striatum, and subsequent dysfunction of dopaminergic and glutamatergic signaling. These effects can contribute to the manifestation of negative symptoms [9].

In a model of neurodevelopmental and neurodegenerative trauma associated with negative symptoms of schizophrenia, it has been observed that an exaggerated stress response can lead to brain damage caused by increased excitotoxicity and oxidative stress [10]. The severity of negative symptoms in patients with schizophrenia may be strongly associated with the extent of oxidative stress damage, which can involve interactions among various oxidative stress markers [11, 12]. Furthermore, administration of antioxidants has been shown to significantly improve the negative syndrome of schizophrenia patients [13].

Sulforaphane (SFN) is an isothiocyanate compound, chemically known as 1-isothiocyanato-4-(methylsulfinyl)-butane. It has been proven to possess both anti-inflammatory and antioxidant effects. SFN is considered the most promising isothiocyanate substance with anticancer properties, and is regarded as the potent anticancer ingredient found in vegetables to date. It is widely consumed as part of the dietary supplements [14]. SFN exhibits its effectiveness through multiple mechanisms, with current research focusing on its anti-inflammatory and antioxidant effects. The antioxidant mechanism of SFN is attributed to its ability to induce the expression of heme oxygenase 1 (HO-1) through the kelch-like ECH-associated protein l (Keap1)/nuclear transcription factor E2-related factor 2 (Nrf2)/antioxidant response element (ARE). Additionally, SFN reduces levels of reactive oxygen species (ROS) in mitochondria, thereby promoting the production of cellular superoxide dismutase (SOD) and glutathione (GSH) [15]. SFN increases the levels of antioxidant enzymes such as SOD and catalase, through the activation of NrF2 and decreases the cellular ROS and prevents apoptosis, which protects human cells from oxidative stress. SFN can enhance the expression level of SOD at not only at the mRNA level but also at the protein level [16]. The anti-inflammatory activity of SFN is mainly based on the inhibition of the nuclear factor kB (NF-kB) pathway, resulting in decreased NF-kB expression and IkB phosphorylation levels. Cyclooxygenase, nitric oxide synthase, tumor necrosis factor-alpha (TNF-α), and IL-6 are also decreased [17]. SFN significantly reduces the serum triglycerides and CRP induced by a high-fat diet, increased high density lipoprotein (HDL) cholesterol and GSH levels, and normalized aortic SOD. Incorporating cauliflower sprouts into the daily diet of overweight individuals had a notable impact on interleukin-6 (IL-6) and CRP levels, effectively reducing chronic inflammation [18].

Currently, there is limited research examining the potential of SFN to improve negative symptoms in patients with schizophrenia. In particular, few studies have investigated the impact of SFN on oxidative stress and C-reactive protein (CRP) levels in schizophrenia patients showing improvement in negative symptoms. Therefore, this study aims to explore the correlation between the effectiveness of SFN in ameliorating negative symptoms in schizophrenia patients and the levels of oxidative stress and CRP.

## Subjects and methods

Patients with schizophrenia were enrolled in the Brain Hospital of the Affiliated Guangzhou Medical University between October 2019 to January 2021. A total of 66 patients with schizophrenia, ranging in age from 18 to 50 were recruited. This study was approved by institutional review board and informed consents were obtained from the participants or their guardians. Clinical trial identification: NCT03451734.

The inclusion criteria were as follows: (1) outpatients or inpatients who meet the diagnostic criteria of the fifth edition of the Diagnostic and Statistical Manual of Mental Disorders (DSM-5) for schizophrenia; (2) aged 18–50 years old, with at least one guardian to monitor and care for the patient for one year; (3) schizophrenia patients who are in the stable period and predominantly have negative symptoms, have no obvious positive symptoms during the PANSS baseline period and exclude that the improvement of negative symptoms is caused by the improvement of positive symptoms, such as hallucinations and delusions; (4) within two weeks prior to the assessment, at least one item should be scored ≥ 4 in all PANSS negative syndrome items (N1 Affective dullness, N2 affective withdrawal, N3 affective communication disorder, N4 Passive/apathy/social withdrawal, N5 Abstract thinking concepts, N6 Lack of spontaneity and fluency in conversation, N7 Stereotyped Thinking).

The exclusion criteria were as follows: (1) history of substance dependence or psychotic symptoms caused by other mental diseases; (2) having traumatic brain injury, epilepsy, or other organic brain diseases; having a suicide attempt or suicidal thoughts; (3) laboratory tests show liver and kidney dysfunction or other serious physical diseases; pregnant or breastfeeding patients; those who are allergic to SFN.

This study used the following withdrawal/stop criteria: (1) subjects with serious adverse drug reactions; (2) switching drugs or treatments due to unstable conditions; (3) refusing to continue to participate in this study; (4) subjects meeting exclusion criteria (such as physical illness, pregnancy, etc.) during the intervention period.

### Intervention and follow-up

Prior to their involvement in this study, all patients had been receiving a stable dosage of antipsychotic medication for at least three months. The antipsychotic treatment regimen remained unchanged throughout the study, guaranteeing that any alterations in negative symptoms observed after the baseline period were induced by the combined treatment with SFN. Patients were administered a standard combination tablet of SFN (Nutramax, produced by Nutramax Laboratories Consumer Care) at a dosage of 850 mg*3 tablets per day (each tablet contained 30 mg of SFN) for 24 weeks.

Participants were provided with a form and instructed to document their daily intake of SFN. These forms were reviewed and assigned a compliance score to evaluate the participants' adherence to the treatment during each visit. Clinical interviews were conducted every 12 weeks to assess the participants' medical condition. Blood tests and TESS scale tests were conducted at baseline, as well as at weeks 12 and 24, to ensure the safety of SFN.

To control variables such as medication adherence, physical exercise, and lifestyle, participants were required to fill out and upload an adverse reaction form every week under the supervision of their families. The research team monitored and reviewed this process. The participants and their families were fully informed and instructed that short-term discomfort during the SFN treatment may occur in the early stage, and most of them can be relieved by themselves and can also be treated accordingly if necessary.

Physicians conducted weekly follow-ups to gather information regarding adverse events, including their type and severity, onset time, duration, and any medications used to address them. Additionally, patients had the option to contact their doctor via WeChat or phone at any time for guidance and could request the discontinuation of treatment if they found it intolerable. In cases where the patients' condition fluctuated, both the patients and their families were promptly notified to seek timely medical attention. Detailed information on the patients' specific conditions was collected and analyzed.

If a patient was unable to continue participating in the study, they were allowed to withdraw from the study at any time. Three clinical follow-up visits were conducted at the baseline, 12-week, and 24-week time points. These visits included physical examinations, electrocardiograms, blood tests, PANSS scale assessments, TESS scale evaluations, assessment of medication dosage, and monitoring of medication compliance.

### Assessments and efficacy indexes

The negative subscale of the PANSS was used to assess the negative symptoms of enrolled patients, with higher scores indicating more severe symptoms. All graders were psychiatrists well trained, and the correlation coefficient for PANSS was greater than 0.80 between graders. The TESS was used to assess whether patients had drug side effects. TESS has the most complete items and the widest coverage among similar scales, including not only common adverse symptoms and signs, but also several experimental results. Efficacy indexes: To investigate whether the significant changes in SOD and HsCRP after treatment were related to SFN, the patients were further divided into different groups based on efficacy according to the reduction rate of the PANSS negative subscale. PANSS negative syndrome reduction rate = (baseline total score- total score after 24 weeks of treatment)/(baseline total score-7) × 100%. If the score reduction rate was equal to or greater than 25%, it was considered to be responder [19].

### Statistical analysis

Data were evaluated using the IBM SPSS 26 statistical program. The *t*-test was used for data that conformed to a normal distribution, and the Wilcoxon rank-sum test was used for data that did not conform to a normal distribution. Chi-square test was used to compare differences in gender and education level; repeated measures analysis of variance was used to analyze the index changes of PANSS negative subscale, SOD, HsCRP, blood routine, liver function, renal function, blood lipids, blood glucose, and myocardial enzymes. The two-way repeated measures analysis of variance was used to analyze the changes of SOD and HsCRP in patients grouped based on the therapeutic efficacy indicated by the PANSS negative subscale. A repeated measures analysis of variance was performed to conduct a significant multivariate test, followed by a univariate analysis to examine individual effects. Specifically, if the interaction between the group and time factors was not significant, no further statistical tests were conducted. If the interaction was significant, we conducted a covariance analysis with baseline scores as covariates to compare between-group differences at the 12-week and 24-week time points. The results were shown as mean ± standard deviation (SD) or median with interquartile range (IQR). Impacted by COVID-19 in early 2020, thirteen subjects were missing the second follow-up, and the missing values were adjusted by the method of mean completer. The comparisons between time points within groups were corrected using Bonferroni.

## Results

### Demographic and clinical characteristics of patients

A total of 66 outpatients who met the DSM-5 diagnostic criteria for schizophrenia and were in a stable period (no fluctuation in the disease condition in the past two months), were recruited from the Brain Hospital Affiliated to Guangzhou Medical University, and their original antipsychotic treatment was continued. Antipsychotics used were as follows: amisulpride (*n* = 24), olanzapine (*n* = 14), risperidone (*n* = 14), quetiapine (*n* = 3), aripiprazole (*n* = 3), paliperidone (*n* = 3), blonanserin (*n* = 2), clozapine (*n* = 1), ziprasidone (*n* = 1), perospirone (*n* = 1). The mean duration of participants taking their current medication was 68.26 ± 73.33 months, and the daily dose was 467.78 ± 164.5 mg (chlorpromazine equivalent).

**Fig. 1:**
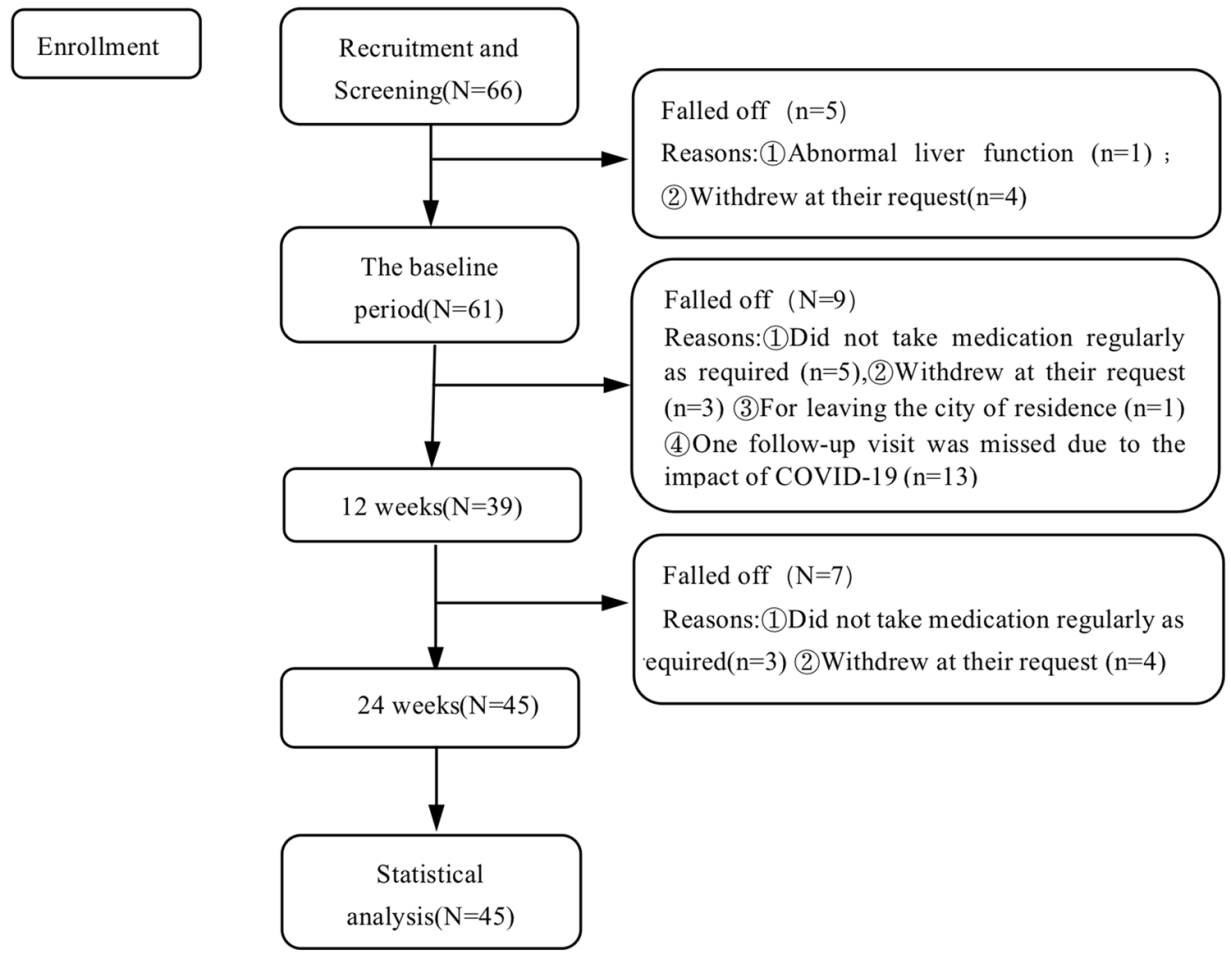
Study sample flowchart

Of the 66 patients (Fig. 1), four patients withdrew their consent for personal reasons before the first assessment, and one patient withdrew from the study after the first assessment with significantly abnormal liver function. A total of 61 patients completed the follow-up in the baseline period. Nine patients withdrew during the second trial due to the following reasons: five patients did not take their medication regularly as required; three patients withdrew at their request; one patient was unable to continue follow-up due to leaving the city of residence. Thirteen patients missed a follow-up due to the impact of COVID-19 in early 2020, and 39 patients completed the second follow-up. Seven patients withdrew during the third trial because three did not take their medication regularly; four self-requested. Finally, 45 patients completed the third follow-up. There was no evidence that participants dropped out of the study due to SFN-related adverse events, with a completion rate of 73.77%. Among the 45 patients, 27 were males and 18 were females, with an average age of 26.33 ± 8.87 (16–50 years old), 14 cases received primary and junior high school education, 18 cases received high school education, and 13 cases had university educations. The average disease duration was 68.26 ± 73.33 months.

### Sulforaphane effects on psychiatric symptoms

As shown in Table 1, the combined treatment with SFN significantly improved the PANSS negative syndrome total score, and the five subscales of N1, N2, N3, N4, N5 (all *P*_Bonferroni_ < 0.001). There was also a significant improvement in the PANSS total score (*P*_Bonferroni_ < 0.001). However, there was no statistically significant change in the PANSS positive subscale (*P*_Bonferroni_ > 0.05).

**Table 1:** PANSS negative subscale and negative subscale analysis results of three follow-up visits (mean ± SD)

	Baseline	12 weeks	24 weeks	F	*P*
PANSS total	63.67 ± 13.84	54.21 ± 13.85	51.67 ± 10.34	25.47	< 0.001
PANSS Positive	11.29 ± 4.57	10.14 ± 3.91	10.18 ± 3.71	2.74	0.83
PANSS Negative	21.04 ± 5.09	17.74 ± 4.69	15.98 ± 4.17	3.14	< 0.001
N1 Blunted affect	3.09 ± 0.95	2.63 ± 0.87)	2.47 ± 0.73	14.41	< 0.001
N2 Emotional withdrawal	3.64 ± 1.07	3.02 ± 0.92	2.51 ± 0.87	41.59	< 0.001
N3 Poor rapport	2.93 ± 1.07	2.53 ± 0.8	2.29 ± 0.69	8.03	< 0.001
N4 Passive/apathetic social withdrawal	3.98 ± 1.03	3.27 ± 0.86	2.71 ± 0.82	27.41	< 0.001
N5 Difficulty in abstract thinking	2.64 ± 1.46	2.12 ± 1.27	2.13 ± 1.22	8.60	< 0.001
N6 Lack of spontaneity and flow of conversation	2.82 ± 1.17	2.44 ± 1.06	2.27 ± 1.05	4.70	0.01
N7 Stereotyped thinking	1.93 ± 0.81	1.72 ± 0.84	1.6 ± 0.84	3.44	0.04

Multiple comparisons were performed on the 7 sub-items of negative symptoms of PANSS with Bonferroni correction

PANSS = Positive and Negative Syndrome Scale

### The correlation of SFN efficacy with SOD and HsCRP

**Fig. 2:**
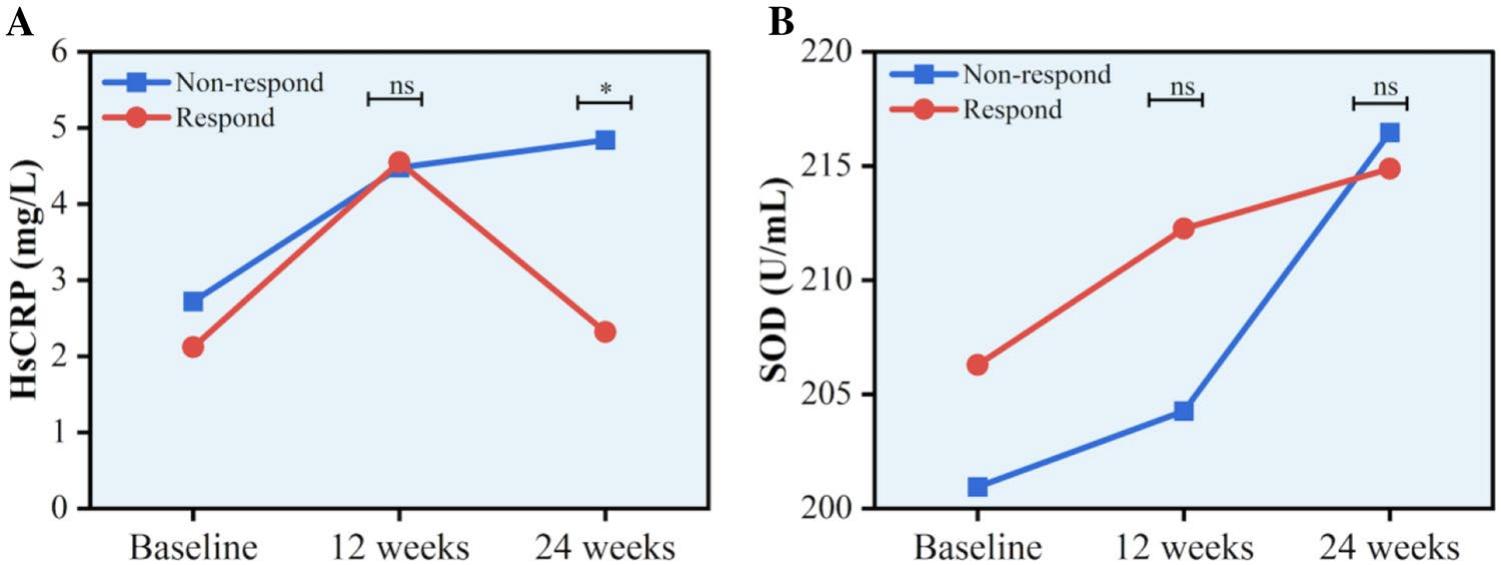
The correlation of SFN efficacy with SOD and HsCRP. ns: Not statistically significant. *P < 0.05. **A** There was no significant difference in the improvement of HsCRP negative symptoms between the responders and the non-responders at 12 weeks (*F* = 0.013, *P* > 0.05), and there was a significant difference between the two groups at 24 weeks (*F* = 7.754, *P* < 0.05). **B** There was no interaction of SOD between treatment group and time (*F* = 0.068, *P* > 0.05)

Two-way repeated ANOVA showed CPR sphericity test < 0.001; multivariate test was chosen and the results were corrected using the Greenhouse–Geisser method; SOD sphericity test = 0.06, which met the sphericity test, and within-subjects effects test was chosen. After combined SFN treatment, the levels of SOD and HsCRP were increased significantly (both *P* < 0.05). The patients were grouped according to the reduction rate of the PANSS negative subscale. There was no significant difference between the responders and non-responders in terms of gender, age, course of the disease, education, SOD, and HsCRP at baseline. As shown in Table 2, there was significant difference in the interaction between** g**roup and time on HsCRP levels (F = 4.34, P = 0.02). Then ANCOVA adjusting baseline HsCRP levels showed that there was no difference in HsCRP levels between responders and non-responders (F = 0.01, P > 0.05) at week 12. The improvement in HsCRP of responders was superior to non-responders at week 24 (*F* = 7.75, *P* = 0.008, *P*_Bonferroni_ = 0.016) (Fig. 2A). As shown in Table 2, there was no significant difference in the interaction between** g**roup and time on SOD levels (*F* = 0.068, *P* > 0.05) (Fig. 2B).

**Table 2:** Comparing the changes of SOD and HsCRP before and after treatment in PANSS negative subscale efficacy grouping (mean ± SD)

	Baseline		12 weeks		24 weeks		*F*	*P*
	Non-responders	Responders	Non-responders	Responders	Non-responders	Responders		
SOD	200.94 ± 23.00	206.29 ± 17.46	204.27 ± 19.66	212.26 ± 20.65	216.46± 18.88	214.88± 19.44	0.70	0.49
HsCRP^a^	2.72± 2.29	2.12 ± 2.30	4.48 ± 4.28	4.55 ± 14.19	4.84± 4.27	2.32± 2.46	4.34	0.02

PANSS score reduction rate ≥ 25% is responder, < 25% is non-responder

Responders (*n* = 28), non-responders (*n* = 17)

SOD = superoxide dismutase, HsCRP = hypersensitive C-reactive protein

^a^Results reported with a correction (Greenhouse–Geisser)

### Safety of SFN

In order to evaluate the safety of the use of SFN, we monitored through routine blood tests, liver function, renal function, blood sugar, blood lipids, myocardial enzymes, and other test indicators, as shown in Table 3, and found no significant changes in blood tests, renal function, and blood sugar, LDH and CK, and the ECG and TESS scale. There were differences in the levels of serum α-hydroxybutyrate dehydrogenase, CK-MB, triglycerides, but did not survive after Bonferroni correction.

**Table 3:** Analysis of the results of three tests at baseline, 12 weeks and 24 weeks of combined sulforaphane treatment (mean ± SD)

	Baseline	12 Weeks	24 Weeks	*F*	*P*
WBC	6.57 ± 1.28	6.74 ± 1.41	6.93 ± 1.89	1.45	0.25
RBC	4.86 ± 0.54	4.86 ± 0.52	4.94 ± 0.53	2.95	0.61
HB	141.6 ± 15.2	141.3 ± 14.65	142.9 ± 15.57	1.09	0.34
TC	4.68 ± 0.84	4.71 ± 0.80	4.68 ± 0.82	0.24	0.79
TG	1.79 ± 1.18	1.70 ± 0.98	1.45 ± 0.85	3.34	0.05
HDL	1.26 ± 0.32	1.21 ± 0.29	1.19 ± 0.26	2.5	0.09
LDL	2.66 ± 0.62	2.72 ± 0.60	2.69 ± 0.52	0.77	0.47
Apolipoprotein A	1.33 ± 0.33	1.22 ± 0.29	1.12 ± 0.19	18.97	< 0.001
Apolipoprotein E	2.70 ± 0.50	2.78 ± 0.52	2.86 ± 0.59	1.49	0.24
AST	21.96 ± 12.7	21.32 ± 10.96	21.56 ± 11.8	0.57	0.95
LDH	167.10 ± 35.5	165.8 ± 31.13	178.47 ± 41.2	2.87	0.67
HBDH	123.80 ± 33.70	121.74 ± 24.2	134.6 ± 29.12	4.08	0.02
CK	156.80 ± 199.30	138.0 ± 135.6	121.7 ± 65.51	1.16	0.32
CK-MB	13.73 ± 16.29	12.68 ± 8.63	14.21 ± 6.35	3.67	0.03
Urea	4.00 ± 0.85	4.01 ± 0.96	4.10 ± 0.89	0.41	0.66
Creatinine	67.75 ± 18.95	69.29 ± 15.47	70.07 ± 14.83	0.68	0.51
Uric acid	397.87 ± 86.72	391.6 ± 100.0	391.9 ± 84.69	0.18	0.82
B2 microglobulin	1.53 ± 0.34	1.93 ± 263.0	1.60 ± 0.46	1.11	0.34
ALT	30.80 ± 25.57	29.19 ± 24.72	29.87 ± 24.13	0.16	0.85
GGT	32.84 ± 29.85	33.31 ± 28.74	37.84 ± 44.68	0.77	0.47
GLDH	7.23 ± 13.47	4.87 ± 3.90	5.69 ± 4.49	4.25	0.05
Amylase	60.60 ± 18.23	57.00 ± 16.84	57.0 ± 16.75	4.28	0.02
FBG	5.27 ± 0.68	5.23 ± 0.75	5.17 ± 0.84	0.26	0.77
Glycosylated hemoglobin	5.06 ± 1.08	5.29 ± 0.49	5.40 ± 0.40	1.92	0.16
β-HB	0.07 ± 0.06	0.10 ± 0.12	0.08 ± 0.05	1.91	0.16
Insulin	12.23 ± 5.87	12.67 ± 7.08	13.2 ± 8.91	0.26	0.77
C-Peptide	2.21 ± 0.85	2.55 ± 1.16	2.92 ± 3.22	2.45	0.98

The single-factor repeated analysis of variance was performed on all items, and the data that did not conform to the normal distribution were analyzed after logarithmic transformation. *P* < 0.05 was considered statistically significant

WBC = white blood cell, RBC = red blood cell, HB = hemoglobin, TC = total cholesterol, TG = triglyceride, HDL = high density lipoprotein, LDL = low density lipoprotein, AST = aspartate transaminase, LDH = lactic dehydrogenase, CK = creatine kinase, CK-MB = creatine kinase, MB, ALT = alanine aminotransferase, GGT = γ glutamyl transferase, GLDH = glutamate dehydrogenase, FBG = fasting blood glucose, β-HB = β-hydroxybutyric acid, HBDH = α-hydroxybutyrate dehydrogenase

We compared the efficacy and test results of SFN on men and women and found that there was no significant difference between men and women in response to SFN treatment. Besides, in this study, 24 patients were treated with amisulpride, which mainly improved negative symptoms at low doses. In order to exclude the influence of the drug on the study, a two-way repeated analysis of variance (*F* = 1.80, *P* = 0.35) was performed on the PANSS negative syndrome subscale in the amisulpride group and the non-amisulpride group, and no statistical difference between the two groups in improving the negative symptoms, suggesting that the use of amisulpride does not affect the results of SFN improving negative symptoms.

## Discussion

Despite the unclear pathophysiological mechanisms of schizophrenia, there is evidence to suggest that oxidative stress and inflammation may be involved in the disease process [6, 20], and that these factors are associated with the severity of negative symptoms in individuals with schizophrenia [11, 21, 22]. It has been widely demonstrated that SFN exhibits antioxidant and anti-inflammatory effects [23–25]. Previous studies have demonstrated that SFN has a protective effect against phencyclidine (PCP)-induced cognitive impairment and oxidative stress, as evidenced by its ability to attenuate PCP-induced cognitive deficits in schizophrenia-like mice. Additionally, subchronic administration of SFN improved PCP-induced cognitive dysfunction in mice, indicating that SFN may also be effective in improving cognitive function in patients with schizophrenia [26]. Furthermore, in a multicenter randomized controlled study involving 172 patients with first-episode schizophrenia over a period of 22 weeks, SFN was found to improve scores in spatial working memory, reasoning-problem solving, and verbal learning, suggesting that SFN may have a positive impact on specific areas of cognitive function [27].

Several previous studies have failed to demonstrate an improvement in negative symptoms of schizophrenia with SFN [27–29]. Considering that oxidative stress and inflammation are more closely associated with negative symptoms of schizophrenia, we specifically enrolled patients with at least one PANSS negative symptom score greater than 3 at baseline. Our study results revealed a significant reduction in PANSS negative subscale scores. We also ensured that patients with predominantly negative symptoms were enrolled after a 3-month stabilization period and their antipsychotic regimen remained unchanged after enrollment. This approach helped to establish that any changes in negative symptoms after the baseline period were a direct result of the combined SFN treatment effect.

Furthermore, our study results indicate that SFN may improve negative symptoms in schizophrenia patients by exerting anti-inflammatory effects. The current genetic–inflammatory–vascular hypothesis of schizophrenia suggests that chronic systemic inflammation can lead to local microvascular damage in the central nervous system (CNS), which disrupts the blood–brain barrier and impairs the regulation of CNS blood flow homeostasis [30]. Several studies have reported abnormal immune responses in patients with schizophrenia, which suggests that inflammatory and immune responses may play a significant role in the pathogenesis of schizophrenia, and are strongly associated with negative symptoms of the disorder [22, 23]. C-reactive protein (CRP) is a non-specific marker of the inflammatory process, and studies have shown that CRP levels are higher during the onset period of schizophrenia, but normal in non-psychotic states [23]. Moreover, several studies have reported a positive correlation between serum CRP levels and negative symptoms, with patients with schizophrenia exhibiting more severe symptoms [31–33]. Lopez-Chillon et al. reported that SFN had an ameliorating effect on CRP levels [18]. In our study, we also observed significant changes in both SOD and HsCRP levels in the serum of patients treated with combined SFN. We further divided the patients into responders and non-responders, and found a significant improvement in HsCRP levels in the responders compared to the non-responders, based on the PANSS negative subscale reduction rate. HsCRP levels in the responders gradually returned to normal after SFN treatment, while they continued to rise in the non-responders.

There are several factors can affect HsCRP levels, including elevated CRP levels associated with negative symptoms of chronic schizophrenia [34], as well as some antipsychotic medications [35]. Other factors, such as smoking, were not controlled for in our study and may have contributed to the rise in CRP levels. Although HsCRP levels increased in both groups after treatment, a repeated measures analysis of variance showed a significant difference between the responders and non-responders. It is possible that the non-responders with improved negative symptoms responded poorly to SFN, leading to a lack of significant improvement in inflammation and resulting in a further increase in CRP levels. However, our study did not include a control group to definitively show that SFN has an ameliorating effect on negative symptoms in patients with schizophrenia or to demonstrate that SFN improves negative symptoms through anti-inflammatory effects. Further double-blind randomized controlled studies are needed to confirm our findings in the future.

Our results also showed a significant increase in SOD activity after combined SFN treatment, and previous studies have confirmed that oxidative stress in the early course of schizophrenia may lead to negative symptoms [36]. Accumulating evidence has shown that SOD plays a crucial role in the pathogenesis of schizophrenia [37]. Lower cerebrospinal fluid SOD may be associated with neurocognitive deficits in patients [25]. In addition, a study found a negative correlation between CuZnSOD and negative symptoms of schizophrenia in patients with first-episode schizophrenia [38].SFN, as an antioxidant, has anti-oxidative stress effects, induces the activity of phase II detoxification enzymes and increases the production of SOD [15]. However, in this study, SOD did not show specificity in the improvement of negative symptoms. The possible reason may be the effects of antipsychotics. It has been shown that long-term treatment with antipsychotics may have an impact on oxidative stress markers [39], with decreased superoxide dismutase (SOD) and glutathione peroxidase (GSH-Px) activities in patients with chronic schizophrenia compared to health controls [40]. These findings suggest that SFN may have a synergistic antioxidant effect with antipsychotic drugs and contribute to improving negative symptoms.

Finally, our study found that SFN was safe. Multiple clinical studies have demonstrated that the daily intake of appropriate doses of SFN is safe for long-term or short-term use without severe adverse events [41, 42]. The use of SFN doses is divided into two categories: low concentration (0.5–5 mg/kg) and high concentration (25–50 mg/kg) [43]. We administered a low-dose range of turnip sulfur at 90 mg/day. The main side effects observed in our study were digestive symptoms such as nausea, heartburn, and dizziness, all of which resolved without special treatment. Additionally, routine blood, liver function, kidney function, blood glucose, lipid, and cardiac enzyme tests did not show significant changes.

There are several limitations in this study. Firstly, it was a single-arm study that lacked a control group. Although our results showed improvement in negative symptoms, our conclusions are limited by the lack of a control group and the fact that patients continued to receive antipsychotic medication after inclusion in the study. Secondly, the PANSS negative subscale may have been affected by the inclusion of items that are no longer considered relevant to the negative symptom domain and the lack of assessment of subjective aspects of negative symptoms. We need more extensive studies, including randomized, double-blind, placebo-controlled trials, and assays of potential biomarkers of oxidative stress and inflammation. Additionally, we need to include more valid and well-established negative symptom assessment tools, such as the Clinical Assessment of Negative Symptoms Interview (CAINS) and the Brief Negative Symptom Scale (BNSS) in future studies. Since depressive symptoms also influence the presentation of negative symptoms, Calgary Depression Scale for Schizophrenia (CDSS) should be used to evaluate the severity of Depression in the future study. Finally, since there were no healthy controls in the study, the differences in SOD and HsCRP levels between schizophrenia patients and healthy controls cannot be compared and therefore, further study should be conducted to investigate it.

In conclusion, our study suggests that SFN may serve as a safe and effective adjunctive intervention for improving the negative symptoms of schizophrenia. Our findings also suggest that the anti-inflammatory effect of SFN, as evidenced by the reduction in HsCRP levels, may be a possible mechanism for its beneficial effects. However, further randomized double-blind controlled studies are necessary to validate our results and establish the precise mechanisms of action of SFN in schizophrenia patients.

### Supplementary Information

Below is the link to the electronic supplementary material.

Supplementary file1 (DOCX 17 KB)

## Data Availability

The data that support the findings of this study are available from author Jianfei Zeng and Xiaobing Lu upon reasonable request.
