# Supplementary material for: The association of SOD and HsCRP with the efficacy of sulforaphane in schizophrenia patients with residual negative symptoms
Source: Eur Arch Psychiatry Clin Neurosci. 2023 Sep 20;274(5):1083–92. doi: 10.1007/s00406-023-01679-7 (PMC11226471; doi:10.1007/s00406-023-01679-7)
Supplement: Supplementary file 1 — Supplementary file1 (DOCX 17 KB) [file 406_2023_1679_MOESM1_ESM.docx]

**Supplementary tabe1.** Demographic information and clinical characteristics

| Variable | Data |
| --- | --- |
| Gender (male: female) | 27:18 |
| Age (years) | 26.33±8.87 |
| Education level (JHSAB: SHS: COA) | 14:18:13 |
| Duration of disease (months) | 68.26±73.33 |
| **Chief medication** |  |
| Amisulpride | 24 |
| Olanzapine | 14 |
| Risperidone | 14 |
| Quetiapine | 3 |
| Paliperidone | 3 |
| Aripiprazole | 3 |
| Bonanserin | 2 |
| Clozapine | 1 |
| Peropilone | 1 |
| Ziprasidone | 1 |
| Single medication | 25 |
| Combination medication | 20 |

Date are presented as mean±standard deviation or number.

JHSAB = Junior high school and below; SHS = Senior High School; COA = College or above.

**Supplementary table2.** PANSS negative subscale grouping baseline period comparison

|  | | Mean ± SD or N (%) | | T orX^2^ | P |
| --- | --- | --- | --- | --- | --- |
|  |  | Non-responder  (n=17) | Responder (n=28) |  |  |
| Gender | male | 11 (64.7%) | 16 (57.1%) | 0.25 | 0.43 |
|  | female | 6 (35.3%) | 12 (42.9%) |  |  |
| Education level | JHSAB | 4 (23.5%) | 10 (35.7%) | 1.92 | 0.38 |
|  | SHS | 9 (52.9%) | 9 (32.1%) |  |  |
|  | COA | 4 (23.5%) | 9 (32.1%) |  |  |
| Age | | 57.53±54.41 | 74.79±83.03 | -0.76 | 0.45 |
| Illness duration | | 27.59±10.77 | 25.57±7.60 | 0.74 | 0.46 |
| SOD | | 200.94±23.00 | 206.29±17.48 | -0.88 | 0.38 |
| HsCRP | | 2.70±2.29 | 2.12±2.23 | 0.85 | 0.40 |

JHSAB = Junior high school and below; SHS=Senior High School; COA = College or above.

PANSS = Positive and Negative Syndrome Scale.

SOD = superoxide dismutase; HsCRP = hypersensitive C-reactive protein.

**Supplementary table 3****.** Adverse reactions in percentage of patients

| Adverse reaction | Adverse reactions in percentage of patients | |
| --- | --- | --- |
|  | 12Week（N=45） | 24Week（N=45） |
| Nausea | 8.89% | 2.22% |
| Heartburn | 6.67% | 2.22% |
| Dizziness | 4.44% | 2.22% |
| Diarrhea | 2.22% | 0.00% |
| Vomiting | 2.22% | 0.00% |
| Headache | 2.22% | 0.00% |
